# Rapid Proliferation and Differentiation of a Subset of Circulating IgM Memory B Cells to a CpG/Cytokine Stimulus *In Vitro*

**DOI:** 10.1371/journal.pone.0139718

**Published:** 2015-10-06

**Authors:** Camilo Vásquez, Manuel A. Franco, Juana Angel

**Affiliations:** Instituto de Genética Humana, Facultad de Medicina, Pontificia Universidad Javeriana, Bogotá, Colombia; MRC National Institute for Medical Research, UNITED KINGDOM

## Abstract

Circulating human IgM expressing memory B cells have been incompletely characterized. Here, we compared the phenotype and *in vitro* functional response (capacity to proliferate and differentiate to antibody secreting cells) in response to CpG and a cytokine cocktail (IL-2, IL-6, and IL-10) of sorted naïve B cells, IgM memory B cells and isotype-switched circulating memory B cells. Compared to naïve B cells, IgM memory B cells had lower integrated mean fluorescence intensity (iMFI) of BAFF-R, CD38, CD73, and IL-21R, but higher iMFI of CD95, CD11c, TLR9, PD-1, and CD122. Compared to switched memory B cells, IgM memory B cells had higher iMFI of BAFF-R, PD-1, IL-21R, TLR9, and CD122, but lower iMFI of CD38, CD95, and CD73. Four days after receiving the CpG/cytokine cocktail, higher frequencies of IgM than switched memory B cells—and these in turn greater than naïve cells—proliferated and differentiated to antibody secreting cells. At this time point, a small percentage (median of 7.6%) of stimulated IgM memory B cells changed isotype to IgG. Thus, among the heterogeneous population of human circulating IgM memory B cells a subset is capable of a rapid functional response to a CpG/cytokine stimulus *in vitro*.

## Introduction

B cells (Bc) and the antibodies they produce after becoming antibody secreting cells (ASC) are critical for protecting the individual from pathogens and maintaining tissue homeostasis [1, 2]. Instrumental to the function of Bc is their capacity to differentiate from naïve (non-antigen experienced) to antigen experienced memory Bc (mBc) [1]. This process renders mBc capable of promptly responding to a second encounter with a pathogen and is reflected by their propensity to rapidly proliferate and differentiate *in vitro*, in an antigen independent fashion, to T cell dependent (CD40L) and independent stimuli [3, 4]. As an example of the latter, it has been shown that naïve cells depend on Bc receptor signaling, while mBc are activated by CpG and cytokines, without need for Bc receptor stimulation [5]. In mice, mBc can be derived independently of a germinal center (GC^-^, mostly IgM with low frequency of mutations) or dependently on the germinal center (GC^+^, mostly isotype-switched with high frequency of mutations) [6–9]. It is unclear if an equivalent population of GC^-^ exists among human mBc [1]. In addition to naïve Bc and different subsets of mBc, Bc with innate function (non-antigen experienced) has been described in the blood and spleen of mice and humans [2, 10–12]. In the murine spleen marginal zone Bc have unmutated BCR and are capable of rapidly responding to bloodborne T cell—independent antigens [12]. Recently, in humans, it has been shown that both spleen and circulating IgM mBc contain a populations of cells that differentiates to marginal zone-like Bc, as its mouse counterpart, through a NOTCH2 signaling pathway [10].

Being able to discriminate all subsets of human Bc by their phenotype has been a challenging enterprise since the discovery of CD27 as a marker of mBc [13]. This marker is not an absolute marker of mBc [14] and, to date, at least six mBc subsets have been defined, based on their differential expression of CD27 and IgH isotypes [15]. These diverse subtypes of mBc were characterized for their differential replication history and Ig gene repertoire and somatic mutations in the Ig genes [15]. However, at present, it is uncertain how to phenotypically discriminate human innate Bc [10, 16] from “true” antigen experienced IgM mBc that may share key features (expression of both IgM and IgD and low number of mutations in their Ig genes) [1, 11].

We and others have previously shown that rotavirus specific mBc (detected by flow cytometry with a labeled rotavirus antigen) are enriched in the CD27^+^ IgM^+^ mBc and CD27^-^ mBc [17–20]. Moreover, we have shown that Total (non-antigen specific) and rotavirus -IgM mBc detected with a seven day limiting dilution assay—in which Bc are stimulated to produce antibodies with CpG, a cocktail of cytokines (IL–2, IL–6, and IL–10) and murine fibroblasts (as feeder cells)—are differentially capable of switching *in vitro* to secrete IgG [18]. Notably, this stimulus was optimized to preferentially activate mBc over naïve Bc and to induce the former to differentiate to ASC [17, 18, 21]. In these assays, the median cloning efficiencies of Total IgM^+^ and rotavirus -IgM^+^ mBc were lower than those of the corresponding switched mBc. The functional importance of IgM mBc was evidenced by experiments in which purified IgM mBC transferred to immunodeficient mice infected with rotavirus were capable of switching isotype and of controlling antigenemia and viremia [18].

A comprehensive functional study of mBc subsets must include Bc from different tissues, as exemplified by experiments in which mBc with the same phenotype, but originating from blood or tonsils, respond differently *in vitro* to T cell independent (CpG) and T cell dependent (CD40L) based stimuli [22]. However, a functional comparison of blood mBc subsets to mBc from other organs is hampered by their low numbers in blood. To date, few studies have contrasted the function of IgM and switched circulating mBc [5, 23–25].

Here, we compared the phenotype and *in vitro* functional response of sort purified circulating naïve Bc and IgM and switched mBc in response to CpG and a cocktail of cytokines (IL–2, IL–6, and IL–10) previously described [18]. Compared to naïve B cells, IgM mBc had lower iMIF of BAFF-R, CD38, CD73, and IL-21R, but higher iMIF of CD95, CD11c, TLR9, PD–1, CD122, and CD45RO. Compared to switched mBc, IgM mBc had higher iMFI of BAFF-R, PD–1, IL-21R, TLR9, and CD122, but lower iMFI of CD38, CD95, and CD73. Higher frequencies of IgM mBc than switched mBc—and these in turn greater than naïve cells—proliferated and differentiated to ASC four days after receiving the CpG/cytokine cocktail. At this time point after the stimulus a small percentage (7.6%) of IgM mBc changed isotype to IgG. We conclude that among the heterogeneous population of circulating IgM memory Bc, a subset is capable of a rapid functional response *in vitro* to a CpG based polyclonal stimulus. These functional studies may aid in discriminating IgM Bc subpopulations.

## Materials and Methods

### Flow cytometry analyses

This project was approved by the ethics committee of the School of Medicine of Pontificia Universidad Javeriana FM-CIE-5166-10. Healthy adult volunteers signed informed consent forms approved by the ethics committee of the School of Medicine of Pontificia Universidad Javeriana. Ficoll gradient purified PBMCs, were suspended in 1X PBS with 0.5% bovine serum albumin (BSA, Sigma-Aldrich, St. Louis) and 0.02% sodium azide (Mallinckrodt Chemicals, Paris, Ky) and stained with monoclonal antibodies (all from BD Bioscience U.S. unless otherwise stated) against (labeled, clone): CD3 (V500, UCHT1), CD14 (V500, M5E2), CD19 (APC-H7, SJ25C1), CD38 (PerCp-Cy5.5 or AF 700, hit2), CD27 (PE-CF594, M-T271), IgD (VB421, IA6-2), IgM (PerCp-Cy5.5 or AF700, G20-127 and 145–8, respectively), CD40 (PeCy7, 5C3), IL-2Rβ /CD122 (PE, Mik-β3), TLR9 (PE, eB72-1665), CD95 (PE, DX2), CD73 (PE, AD2), CD21 (PE, Bly–4), IL-21R (PE, 17A12), CD45RO (APC, uch1), CD11c (APC, S-HCL–3), BAFF-R (APC, 11C1), CD43 (APC, eBio84-3C1) (eBioscience U.S. San Diego, CA), PD–1 (PE, J105) (eBioscience U.S. San Diego, CA), and CX3CR1 (APC, 2A9-1) (Biolegend, U.S, San Diego, CA), on ice for 30 min [for all the markers analyzed a FMO was include as a negative control (S2 Fig)]. Samples were acquired on a LSR Fortessa (BD Biosciense) and analyzed using FlowJo v.9.3.2. The data are represented as an integrated mean fluorescent intensity (iMFI = percentage of positive cells × MFI), a metric measure that combines magnitude (percentage of cells expressing a marker) and quality (MFI of this same marker) and reflects the total potential functional response of each cell subset [26, 27].

### Sorting of Bc subpopulations

The Hemocentro Distrital, Bogotá, Colombia, provided buffy coats from de-identified healthy donors that signed informed consent forms. From these buffy coats, an enriched suspension of circulating B cells was obtained by negative selection by rosette formation (StemCell Biotech, Vancouver, Canada), as previously described [18]. The enriched B cells were suspended in sterile PBS and stained on ice with AQUA (Invitrogen Molecular Probes U.S. MA, Waltham) and subsequently with antibodies (all from BD Bioscience) against (labeled): CD3/CD14 (V500), CD19 (APC-H7), CD38 (PerCp-Cy5.5), CD27 (PE-CF594), IgD (VB421), IgG (APC G18-145), and IgM (APC polyclonal) (Jackson Immunoresearch U.S. PA, West Grove) for 30 minutes on ice. Samples were sorted using a FACSAria IIu (BD Bioscience) and cell purities were above 95%. Cells were recovered in RPMI supplemented with 2 mM L-glutamine, 100 U/ml penicillin, 100 μg/ml streptomycin, 0.1 mM non essential amino acids, 1 mM sodium pyruvate, 0.05 mM β-mercaptoethanol and 20% Fetal bovine serum (FBS), from now on referred to as complete medium.

### Stimulation and analyses of sort purified Bc

B cells subpopulations were labeled with carboxyfluorescein succinimidyl ester (CFSE), as previously described [28], with minor modifications. Bc were washed twice with sterile PBS and stained (0.4–1 x 10^6^ cells/ml) with CFDA-SE (Cell-TraceTM CFSE Cell Proliferation Kit, Invitrogen Molecular Probes) for 5 min at room temp protected from light. After being washed three times with 10ml of PBS 5%, FBS 5%, naïve Bc and IgM mBc and switched mBc were stimulated, as previously described [18], adapting the limiting dilution assays conditions to stimulation in a 48 well format: Bc were stimulated with 2.5 μg/ml of CpG (ODN 2006; InvivoGen, San Diego, CA), 10 ng/ml human recombinant IL–2, 10 ng/ml human recombinant IL–6, 15 ng/ml IL–10 (all cytokines from PreproTech, Rocky Hill, New Jersey), and NIH 3T3 murine fibroblasts (ATCC Manassas, VA, USA). The NIH 3T3 feeder cells were irradiated with 3,000 rads (Radiotherapy unit, Centro Javeriano de Oncología, Bogotá) and then used at a concentration of 5,000 cells/well. B cells (20,000 in 200 μl of complete medium with 10% FBS) were cultured at 37°C with 5% CO_2_ in flat bottom 48 well plates for different periods of time. At the end of the cultures, cells were washed twice with sterile PBS and stained on ice with AQUA (Invitrogen Molecular probes) and subsequently with antibodies (all from BD Bioscience) against (labeled): CD19 (APC-H7), CD38 (PerCp-Cy5.5), CD27 (PE-CF594). After washing, cells were resuspended in 100 μl PBS, 0.5% BSA (Sigma-Aldrich) plus 250 μl of cytofix/cytoperm (BD Pharmingen) and incubated for 20 min at 4°C. After washing twice with perm/wash (BD Pharmingen) cells were stained with antibodies against IgM APC (Jackson ImmunoResearch) or IgG APC (BD Bioscience) for 30 min on ice. Samples were acquired on a LSR Fortessa (BD Bioscience) and analyzed using FlowJo v.9.3.2 (FlowJo, LLC, U.S. OR).

### Statistical analyses

Since data was not normally distributed, non parametric tests were used for comparisons. Differences among groups were determined using Kruskal-Wallis tests followed by a Wilcoxon signed-rank test using GraphPad Prism software v.5.0a for Mac OS X, GraphPad Software (La Jolla, CA). Significance was established if p<0.05. Data are shown as median and interquartile range.

## Results

### Phenotypic differences of naïve Bc, IgM mBc, and switched mBc

**Fig 1:**
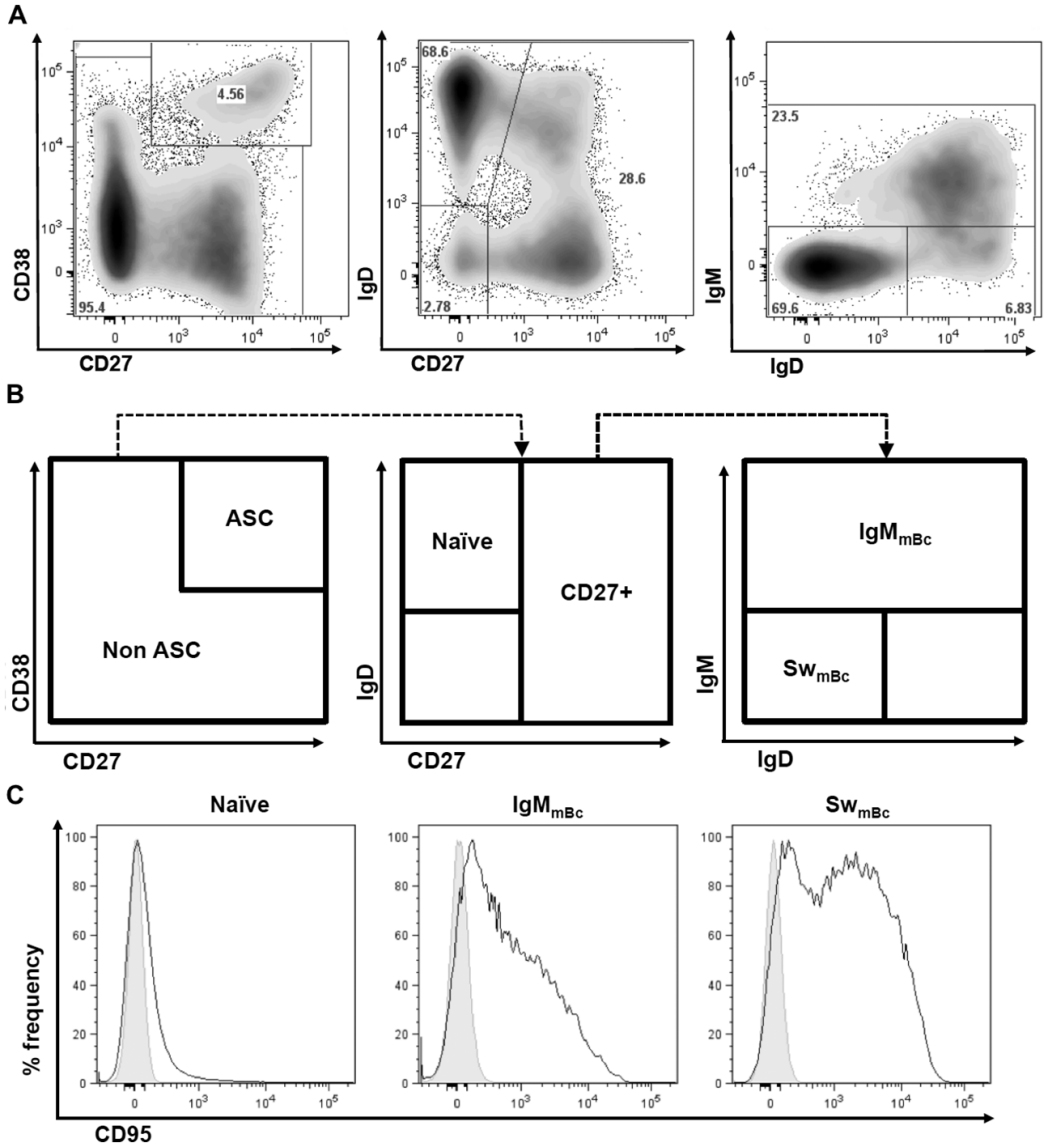
Flow cytometry analysis strategy. Representative dot plots (**A)** and the corresponding strategy (**B**) to identify on a gate of circulating Bc (CD19^+^ CD3^-^/CD14^-^) that excludes CD38^hi^, CD27^hi^ ASC the Bc populations of interest: naïve (CD27^-^ IgD^+^), IgM mBc (CD27^+^ IgD^+^ IgM^+^) and switched (Sw) mBc (CD27^+^ IgD^-^ IgM^-^). Different markers were studied for each Bc subset; shown are representative histograms for CD95. Solid gray histogram represents fluorescence minus one (FMO) and the continuous solid line shows staining with anti-CD95 (**C**).

**Fig 2:**
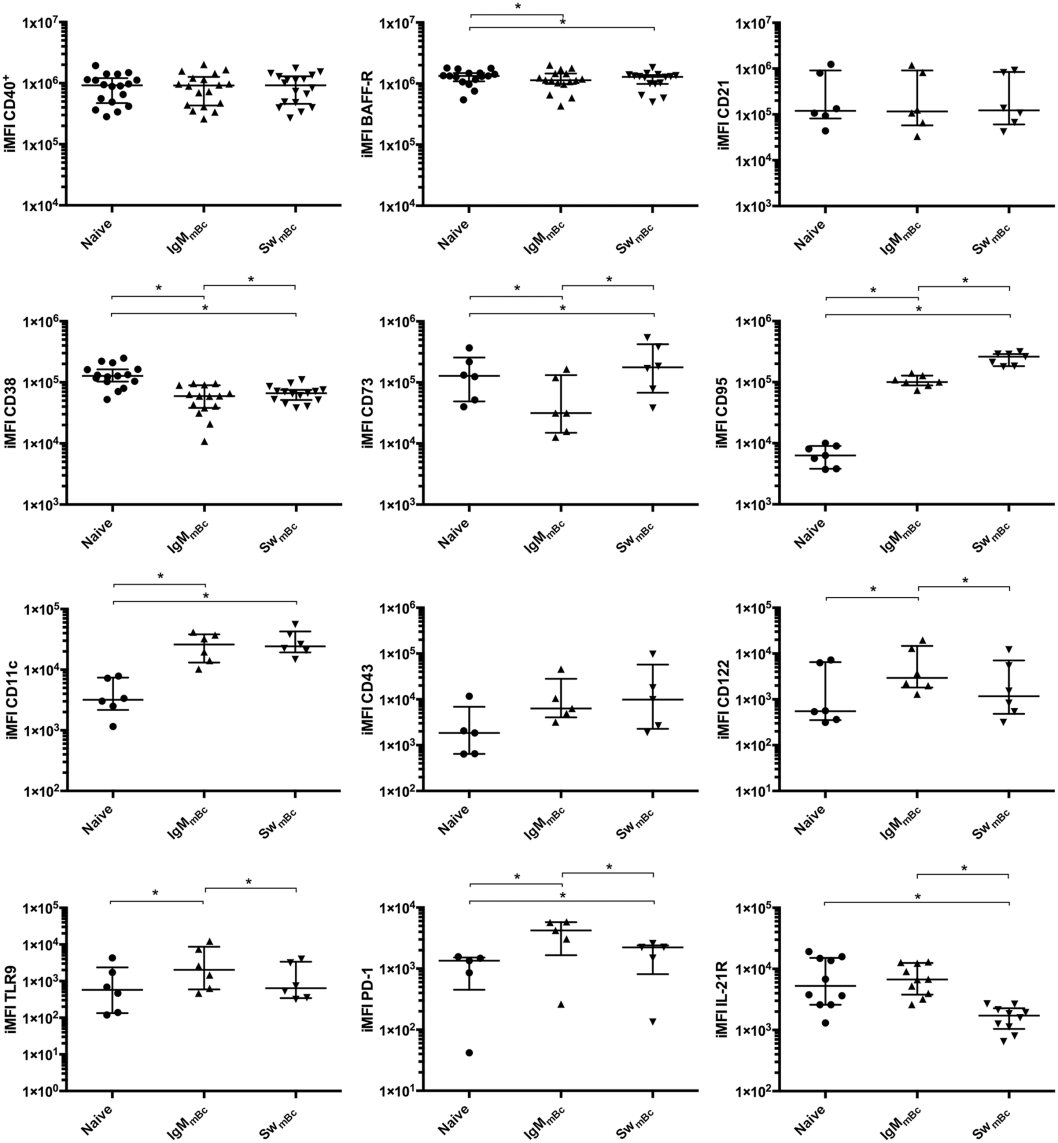
Phenotypic profile of the three Bc subsets. A summary of the experiments to characterize the phenotype of circulating naïve Bc and IgM and switched (Sw) mBc in PBMC from healthy adult volunteers is presented: CD40 (n = 18), BAFF-R (n = 16), CD21 (n = 6), CD38 (n = 15), CD73 (n = 6), CD95 (n = 7), CD11c (n = 6), CD43 (n = 5), CD122 (n = 6), TLR9 (n = 5), PD–1 (n = 5), and IL-21R (n = 10). Wilcoxon tests were used for evaluating differences among groups and * denotes p<0.05. Individual results are shown (as iMFI) and lines and error bars denote the median and interquartile range, respectively.

Bc (CD19+) were separated into naïve (CD27^-^ IgD^+^: the transitional Bc subset was not excluded from the analysis), IgM mBc (CD27^+^ IgD^+^ IgM^+^: which included the IgM only but not the IgD only subpopulations) and switched mBc (CD27^+^ IgD^-^ IgM^-^), as described in Fig 1A and 1B and S1 Fig. For each of the three Bc subpopulations, we evaluated the expression of 12 markers and histograms for one of these (CD95) is shown in Fig 1C, as an example. The selected markers evaluated were: CD40, BAFF-R, CD95, PD–1, IL-21R, CD73, CD43, CD11c, CD21, CD38, TLR9, and CD122. A summary of the experiments is shown in Fig 2 were data is represented as iMFI, a metric measure that combines magnitude and quality. Histograms for each individual marker are shown in S2 Fig.

The iMFI of CD40 and CD21, a part of the Bc co-receptor [29] and considered a molecule highly expressed on marginal zone Bc [30], were comparable among Bc subsets (Fig 2, first row). BAFF-R iMFI was high among Bc and significantly higher in naïve Bc (Fig 2, first row).

The iMFI of CD38, CD73, and CD95 were intermediate to low in the three Bc subsets (Fig 2, second row). CD38 is an ADP-ribosyl cyclase and its iMFI was higher in naïve Bc than both switched mBc and IgM mBc. In agreement with previous results, the iMFI of CD73 (an ecto-enzyme that degrades extracellular nucleoside monophosphates to adenosine) of naïve Bc was comparable to that of switched mBc, but higher than that of IgM mBc [31]. The expression of CD95 is associated with caspase dependent apoptosis [32] and, as previously reported, CD95 iMFI of naïve Bc was comparatively very low [4, 32]. Also, CD95 iMFI of IgM mBc was significantly lower than that of switched mBc.

The iMFI of CD11c a marker shown to be expressed by IgM mBc specific for bacteria in mice [33], was intermediate and similar for IgM and switched mBc, but lower for naïve Bc (Fig 2, third row).

Similar low iMFI of CD43, a marker that has been proposed to identify B1 Bc in humans [16] were identified in the three Bc subset studied (Fig 2, third row).

The iMFI of CD122, the beta chain of the IL–2 and IL–15 receptors, TLR9, the receptor for CpG, and PD–1, which regulates activation and survival of Bc [34], were low for the three Bc subsets, but significantly higher in IgM mBc than naïve Bc or switched mBc (Fig 2, third row and fourth row, respectively). However, both IgM and switched mBc express more intracellular TLR9 than naïve cells, but no differences were seen between IgM and switched mBc (S3 Fig).

Finally, in agreement with previous reports [35], higher iMFI of IL-21R, a key molecule critically implicated in Bc activation [36], was detected for naïve cells than switched mBc (Fig 2, fourth row). We also compared the expression of CD45RO (data not shown), which resembled the expression of CD43 (Fig 2).

### A subset of IgM mBc rapidly proliferate and differentiate to ASCs *in vitro* in response to CpG and cytokines

**Fig 3:**
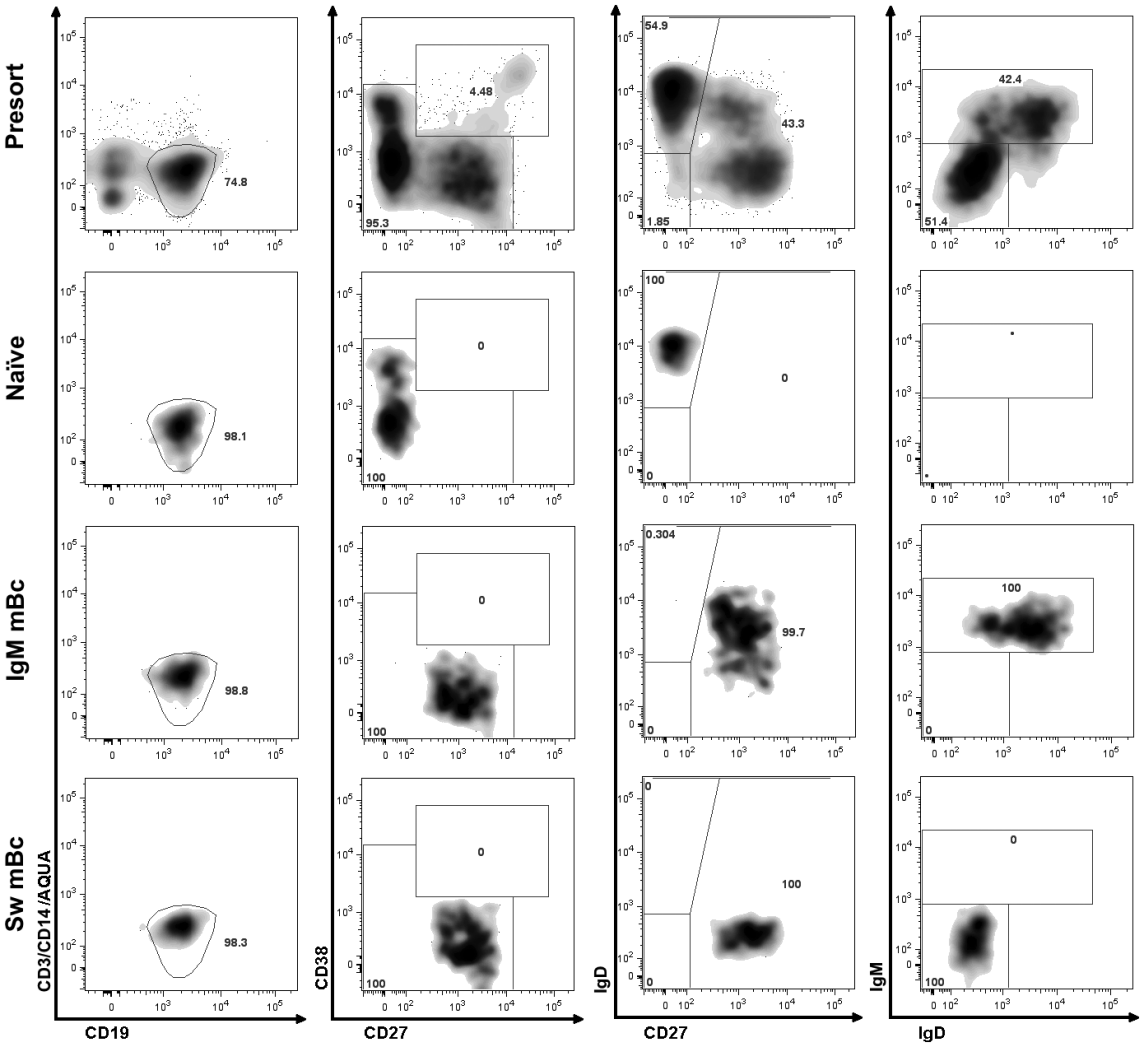
Sorting strategy for purification of the three Bc subsets. Representative dot plots of a sorting experiment to purify naïve Bc and IgM and switched (Sw) mBc. Dot plots of presort samples are shown in top rows. Dot plots of naïve Bc and IgM and Sw mBc are shown in the second, third, and fourth rows, respectively. Cells were initially gated on CD19^+^ dump channel^-^ (Aqua, CD3, CD14), first column. After excluding CD38^hi^, CD27^hi^ ASC (second column), naïve cells were gated as CD27^-^ IgD^+^ (third column) and the CD27^+^ subset was sorted as IgM^+^ or switched (IgM^-^) mBc (fourth column).

**Fig 4:**
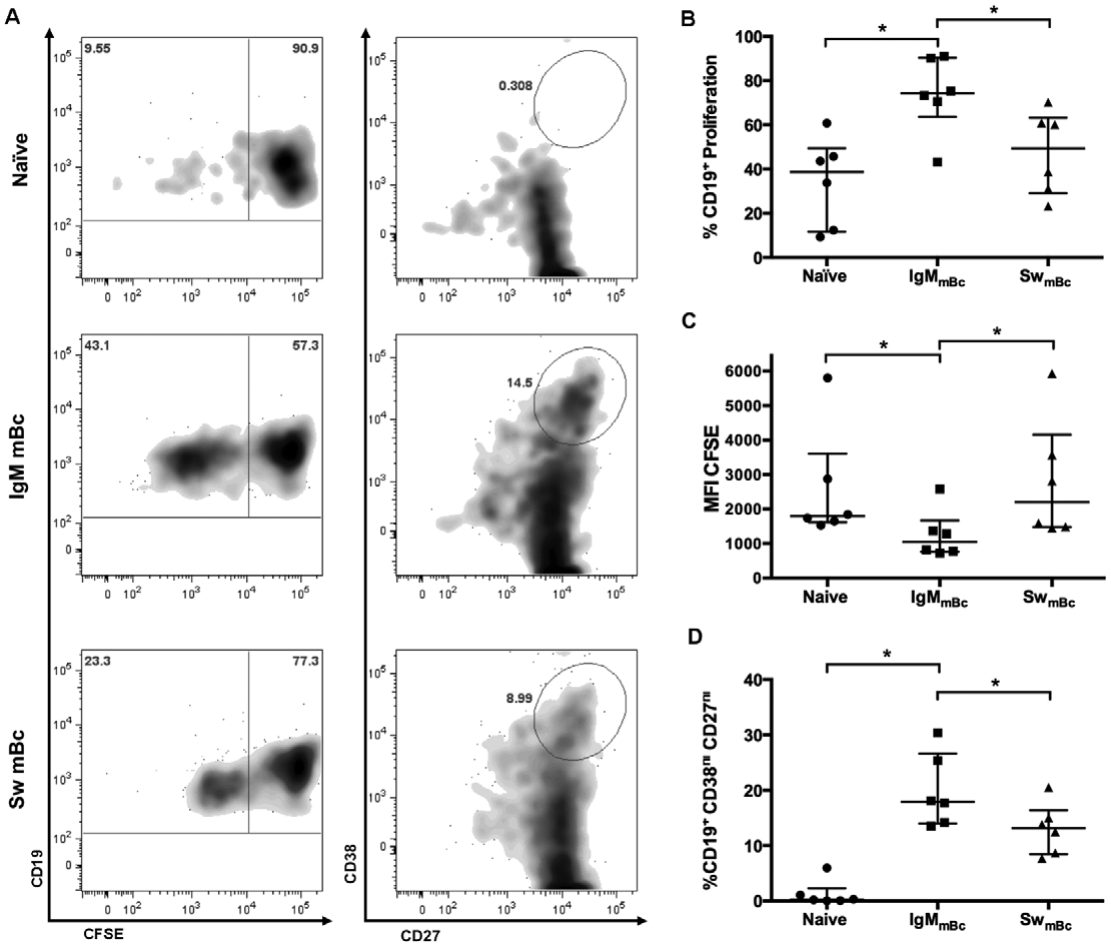
Proliferation and differentiation to ASC of the three Bc four days after *in vitro* stimulation. Representative dot plots of proliferation (first column) and differentiation to ASC (CD38^hi^ CD27^hi^) (second column) of naïve Bc and IgM and switched mBc four days after stimulation with CpG, a cocktail of cytokines (IL–2, IL–6, and IL–10), and murine fibroblasts (as feeder cells) **(A)**. Summary of experiments for percentages of CFSE^low^ proliferating Bc **(B)**, MFI of CFSE^low^ cells **(C)**, and frequencies of CD38^hi^ CD27^hi^ ASC **(D)** of each of the Bc studied. Wilcoxon tests were used for evaluating differences among groups and * denotes p<0.05. Individual results are shown and lines and error bars denote the median and interquartile range, respectively.

To compare the capacity to proliferate and differentiate to ASC (CD27^hi^ CD38^hi^) of naïve Bc and IgM and switched mBc, Bc were enriched from buffy coats of blood bank volunteers by rosette formation and subsequently each subset was purified by the sorting strategy described in Fig 3. Bc were then labeled with CFSE and stimulated with CpG, a cocktail of cytokines (IL–2, IL–6, and IL–10) and murine fibroblasts (as feeder cells) for two, three, four, and five days. The peak response for proliferation and differentiation to ASC (CD27^hi^ CD38^hi^) was between days three and four, for both types of mBc (S4 Fig). Four days after stimulation a higher frequency of IgM mBc proliferated (Fig 4A and 4B) and acquired the phenotype of ASC, compared to switched mBc and naïve Bc (Fig 4D). It has been previously shown that, in general, naïve Bc have undergone a lower number of divisions (two divisions) than IgM mBc (seven divisions) and switched mBc (10 divisions) [15], and that the number of divisions a Bc has performed limits the maximum additional divisions it can undertake [37]. In agreement with this, we found that the MFI CFSE—which is inversely proportional to the number of divisions a Bc has performed *in vitro* after stimulation—was lower in the IgM mBc *vs*. switched mBc (Fig 4C).

### A subset of stimulated IgM mBc rapidly change isotype *in vitro* after stimulation

**Fig 5:**
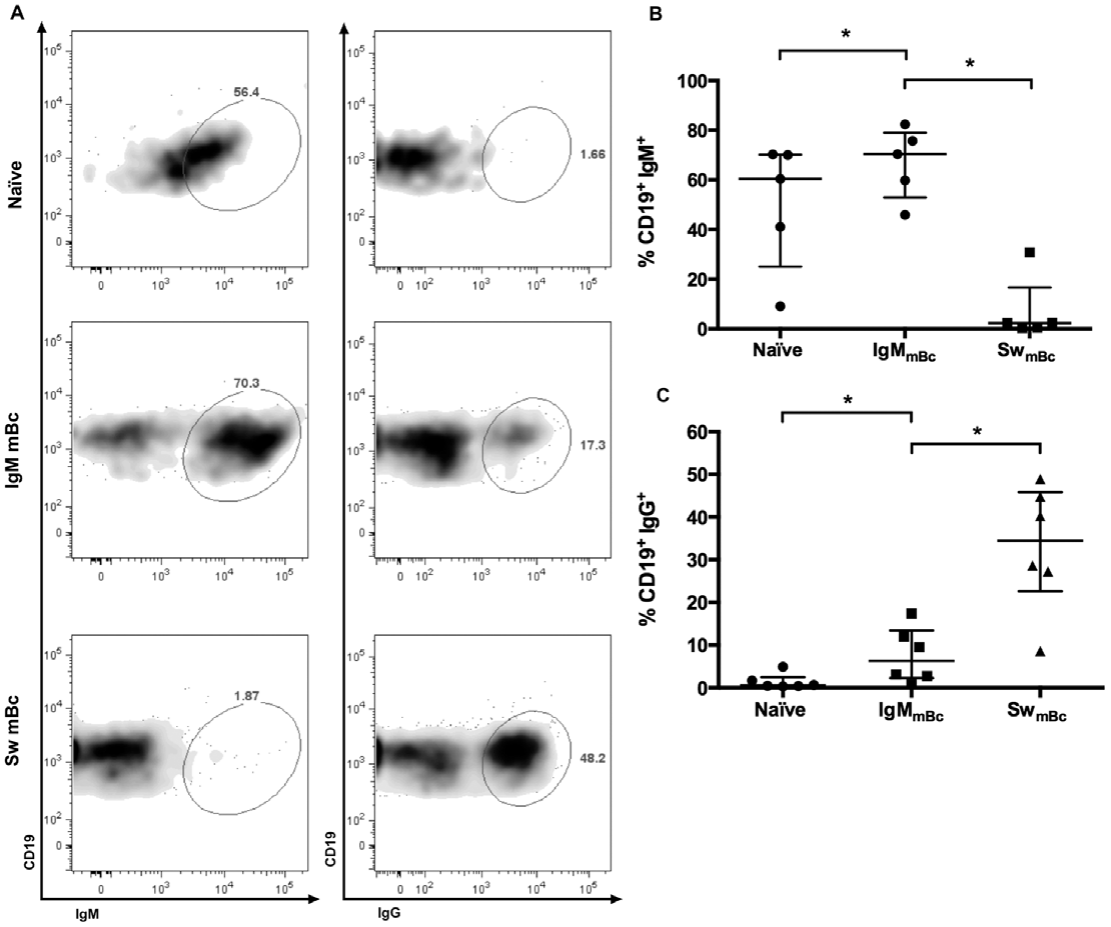
Intracellular expression of IgM and IgG of the three Bc subsets four days after *in vitro* stimulation. Representative dot plots of an experiment to evaluate intracellular IgM and IgG after stimulation of naïve Bc and IgM and switched mBc (**A**). Summary of experiments for the percentages of cells expressing IgM (**B**) and IgG (**C**) are shown. Wilcoxon tests were used for evaluating differences among groups and * denotes p<0.05. Individual results are shown and lines and error bars denote the median and interquartile range, respectively.

Our group has previously shown that a median of 62% IgM mBc stimulated for seven days in a limiting dilution assay, with the same stimulus as we have used here, switch to IgG [18]. To determine if isotype switch occurred in IgM mBc under the present stimulation conditions (four days), we stained Bc for intracellular IgM and IgG (Fig 5A). Coherent with our initial results, we observed an isotype switch to IgG in a median of 7.6% (range 2–20%) of IgM mBc four days after stimulation (Fig 5A and 5C). As expected, very low or no naïve Bc expressed IgG and no, or very low, numbers of switched mBc expressed IgM. Of note the median IgG expression on switched mBc was 34.4% because the subset was sorted as IgD-, IgM- cells that include IgA an IgE cells. Intracellular IgG expression was not found on purified naïve Bc and IgM mBc prior *in vitro* stimulation (S5 Fig).

## Discussion

Comparison of the functional capabilities of switched and IgM mBc may help in better discriminating human Bc subsets. Here, we have shown that four days after stimulation with a CpG/cytokine cocktail (IL–2, IL–6, and IL–10) (Fig 4), a higher frequency of circulating IgM mBc proliferate and differentiate *in vitro* to ASC (CD38^hi^ CD27^hi^) compared to switched mBc and naïve Bc, and a subset of IgM mBc are already capable of switching to IgG (Fig 5). We have also identified/corroborated phenotypic differences between the three Bc subpopulations, which may help explain this functional behavior (Fig 2).

Antigen independent functional response to T cell dependent (CD40L) or independent (CpG) stimuli (in the absence or presence of multiple cytokines) of human spleen naïve Bc and IgM and switched mBc have been extensively compared [3, 4, 38, 39]. In general, these studies have reported important differences between the naïve and mBc, but only minor or no dissimilarities between the two mBc subsets: higher frequencies of IgM and switched spleen mBc differentiate to ASC in response to CD40L, compared to naïve Bc [40]. A greater proportion of human spleen switched mBc than IgM mBc was induced to proliferate after stimulation with CD40L, IL–2, IL–10, and IL–21 [3, 4, 38]. In contrast, and in agreement with our results with blood Bc (Fig 4), when spleen IgM and switched mBc were stimulated with CpG a higher percentage of IgM mBc than switched mBc proliferated [4]. Also, the differentiation rates to ASC of the two spleen mBc have been comparable or slightly different, depending on the cytokine used for co-stimulation [40]. Extrapolation of these results to Bc from other organs has been put in doubt by experiments showing that Bc from human tonsils differ functionally from Bc subsets with the same phenotype isolated from blood [22]. Thus, a comprehensive analysis of B cell subsets must include functional studies of B cells from different organs including blood, like in the present study.

Studies that have compared blood naïve Bc and IgM and switched mBc are scarce: while (in agreement with our results, Fig 4) higher frequencies of blood IgM mBc seem to proliferate in response to CpG, switched mBc appear to better respond to an alloreactive T cell clone [5]. After stimulation with *Staphylococcus aureus Cowan*, IL–2, IL–10, and anti-CD40 for eight days, a similar differentiation of CD27^+^ IgD^+^ mBc (most probably IgM mBc), and CD27^+^ IgD^-^ mBc (most probably switched mBc) to CD38^+^ ASC was observed [23]. More recently, it has been shown that blood IgM mBc (excluding IgM only mBc) share a similar gene expression pattern with switched mBc [25]. Notwithstanding, in response to a BCR stimulus, in the presence or absence of CD40L, IgM mBc up regulate markers that permit them to migrate to B-cell follicles, whereas activated IgG+ mBc preferentially showed a plasma cell differentiation. In this study it was also shown that T-independent stimulus, like terbutaline and CEACAM8, preferentially stimulated IgM mBc, while CD40L (a T-dependent stimulus) preferentially stimulates switched mBc [25]. This last study highlights the plasticity of IgM mBc that respond differently depending on the stimulation protocol. For this reason it is important to note that our results maybe be restricted to our stimulation procedure (based on CpG and cytokines), which was previously standardized for the study of antigen specific mBc [17, 18, 21]. In our previous experiments stimulating purified IgM and switched mBc, but in a limiting dilution assay for seven days, we found a higher cloning efficiency in the latter, which was accompanied, at this time point, by induction of higher frequencies of ASC identified by flow cytometry and ELISPOT [18]. Altogether, these results and our new findings (Fig 4) suggest that IgM mBc proliferate and differentiate more rapidly than switched mBc in response to CpG/cytokines, but that with time a greater proportion of switched mBc can become functional [18]. Finally, in support for a selective effect of CpG on IgM mBc, it has been shown that in blood CD27^+^ mBc CpG induced increased IgM secretion, compared to CD40L [41].

Our results showing that IgM mBc, but not naïve Bc, can change isotype to IgG by day four (Fig 5A and 5B) are coherent with previous studies in which cells were stimulated with CD40L, IL–2, and IL–10 for 10 days [24]. However, in the study that compared CD27^+^ IgD^+^ and CD27^+^ IgD^-^ mBc stimulated with CD40L and IL–2 or SAC plus IL–2 for eight days no isotype switch was seen in the IgD^+^ CD27^+^ (mainly IgM mBc) [23]. Nonetheless, when IL–10 was added to the Bc cultures, IgM mBc were shown to secrete IgG two to three days after stimulation [23]. Altogether, these reports are in agreement with our findings (Fig 5), indicating that IgM mBc can rapidly change isotype to a non BCR stimulus, but that this is dependent on the presence of cytokines like IL–10, similar to what has been reported for spleen IgM mBc [40].

Naïve Bc and IgM and switched mBc were shown to have several phenotypic differences (Fig 2). Compared to switched mBc, IgM mBc had higher iMFI of molecules involved in the activation of Bc, such as TLR9 [5, 41, 42], IL-21-R [43], and CD122 [44] (Fig 2). Of these markers the expression of TLR9 and CD122 may partially explain the higher proliferative and capacity to differentiate to ASC of IgM mBc compared to switch mBc in our assay (Fig 4). Activation through TLR9 has been shown to selectively provoke proliferation of mBc and in particular of IgM mBc [5, 41, 42]. In agreement with our results for surface protein (Fig 2), a tendency to higher expression of TLR9 mRNA has been shown in IgM mBc than in naïve Bc and switch mBc [45]. Although this difference may explain in part the functional differences of IgM and switched mBc (Figs 3, 4 and 5), this interpretation must be taken with precaution because most of this receptor is expressed intracellularly [4] and when we measured intracellular TLR9 no differences were observed between IgM and switched mBc (S3 Fig). Since we included IL–2 in the Bc cultures, higher expression of CD122 (the IL–2 receptor) on IgM mBc could also partially explain the increased proliferation of these cells (Figs 3, 4 and 5). Because we also included IL–10 and IL–6 in our cultures, higher expression of IL-10R but lower expression of IL-6R mRNA by IgM mBc than in switched mBc [25] may also modulate the increased proliferation/differentiation of the former. Compared to switched mBc lower frequencies of IgM mBc express CD95 (Fig 2), which regulates Bc survival [32]. The expression of this marker on mBc may also give them an *in vitro* survival advantage that could be reflected on their increased proliferation frequencies (Fig 4).

The capacity of a cell to switch has been related to the presence of CD40 [46], BAFF-R [47], CD73 [31], IL21R [43] and TLR9 [48]. Of these markers, CD73 seems like a good marker to identify the subset of IgM mBc that switch *in vitro* in response to our stimulus (Fig 5). In favor of this hypothesis, it was recently reported that the presence of CD73 correlated with the capacity to switch isotype of IgM mBc [31]. Moreover, in mice, CD73^+^ mBc are enriched in those with somatic mutations [9] and GC^-^ IgM mBc were mainly CD73^+^ [7]. However, the frequencies of CD73 expressing IgM mBc [31] (Fig 2) are higher than those of cells undergoing isotype switch (Fig 5). Using a combination of CD73 with one or more of the above mentioned differentially expressed markers involved in activation, survival, or isotype switch may be useful to identify IgM mBc that switch isotype after this stimulus.

The nature of human blood IgM^+^ CD27^+^ Bc is a subject of controversy [11, 30]. At least a part of the subset that expresses IgD seem to be innate Bc with a pre-diversified repertoire of Ig [49, 50], which share many markers with spleen marginal zone—and similar to those present in mice—but capable of circulating in blood [10]. Besides, some studies [51], but not other [52], have shown that the CD27^+^ IgM^+^ IgD^+^ Bc are clonally related to switched mBc, suggesting that at least a fraction of these Bc may be true IgM mBc. In support for this hypothesis, CD27^+^ IgM^+^ IgD^+^ cells transferred to immunodeficient mice immunized with *Streptococcus pneumoniae* [53] can develop specific IgG mBc. The existence of human blood IgM^+^ IgD^+^ mBc specific for rotavirus [17, 18], tetanus toxoid [17, 54], and the D antigen [54] also suggest that at least a fraction of CD27^+^ IgM^+^ IgD^+^ Bc are true mBc. Finally, as previously mentioned, IgM mBc (excluding IgM only mBc) share a similar gene expression pattern with switched mBc [25]. Thus, the CD27^+^ IgM^+^ IgD^+^ Bc seem to be heterogenous, a part being innate (related to marginal zone Bc) while other being antigen dependent mBc (probably GC^-^) [11].

In conclusion, we have shown that a subset of IgM mBc rapidly proliferate and differentiate to ASC early after stimulation with CpG/cytokines. The functional assay we have described (Figs 4 and 5), applied to subsets of purified Bc selected based on combinations of the markers that differentiate naïve Bc and IgM and switched mBc (Fig 2), may be useful to identify various subpopulations among the IgM mBc.

## Supporting Information

S1 Fig Initial work-flow and pre-gating for phenotype and sorting experiments. As indicated in the figure, sorting experiments include a negative microbead enrichment step that incorporated microbeads with antibodies against CD3, CD14, CD16, and CD56. Moreover, staining for both phenotype and sorting experiments included a dump channel with antibodies against CD3, CD14, and AQUA.
(TIF)

S2 Fig Phenotypic profile of the three Bc subsets. Representative histograms of the markers studied in naïve Bc (blue), IgM mBC (red) and Sw mBc (green) subsets. Solid gray histogram represents fluorescence minus one (FMO).
(TIF)

S3 Fig Intracellular expression of TLR9. Summary of intracellular TLR9 iMFI of naïve Bc and IgM mBC and Sw mBc (n = 6).
(TIF)

S4 Fig Proliferation kinetics and differentiation to ASC (CD38^hi^ CD27^hi^) of IgM and switched mBc. Kinetic experiment of proliferation and differentiation to ASC (CD38^hi^ CD27^hi^) of IgM (brown) and switched mBc (blue) four days after stimulation with CpG, a cocktail of cytokines (IL–2, IL–6, and IL–10), and murine fibroblasts (as feeder cells) for two, three, four, and five days. Kinetic experiments for early (one, two or three days) and late responses (five, seven and ten days) were also performed using ELISPOT as readout (data not shown). Since the early time point experiments showed comparable results to the flow cytometry presented in this figure and cell mortality was above 80% in the late time point experiments (data not shown), we chose day four for the experiments reported elsewhere in this paper.
(TIF)

S5 Fig Post sort control for intracellular expression of IgG on sorted naïve Bc IgM mBc and Sw mBc. Cells from a buffy coat were sorted like for the functional experiments of Figs 3 and 4 and directly stained for intracellular IgG as described for Fig 5.
(TIF)
