# Supplementary figures and images for: Rapid Proliferation and Differentiation of a Subset of Circulating IgM Memory B Cells to a CpG/Cytokine Stimulus In Vitro
Source: PLoS One. 2015 Oct 6;10(10):e0139718. doi: 10.1371/journal.pone.0139718 (PMC4595470; doi:10.1371/journal.pone.0139718)

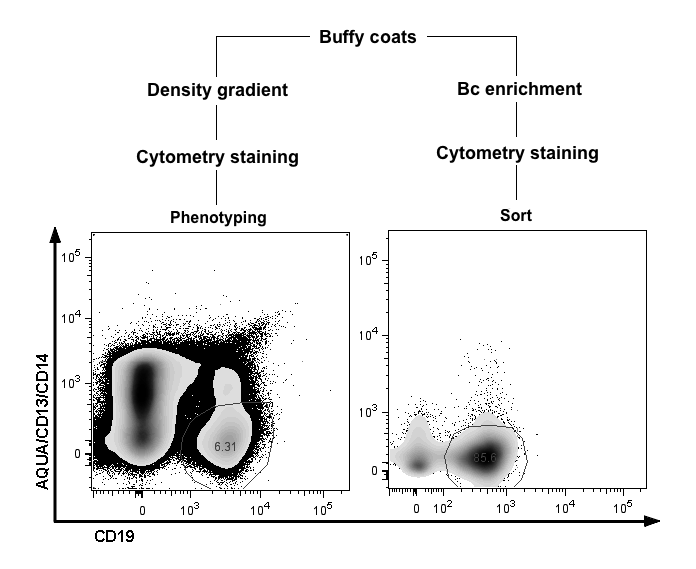

Supplement: S1 Fig — As indicated in the figure, sorting experiments include a negative microbead enrichment step that incorporated microbeads with antibodies against CD3, CD14, CD16, and CD56. Moreover, staining for both phenotype and sorting experiments included a dump channel with antibodies against CD3, CD14, and AQUA. (TIF) [file pone.0139718.s001.tif]

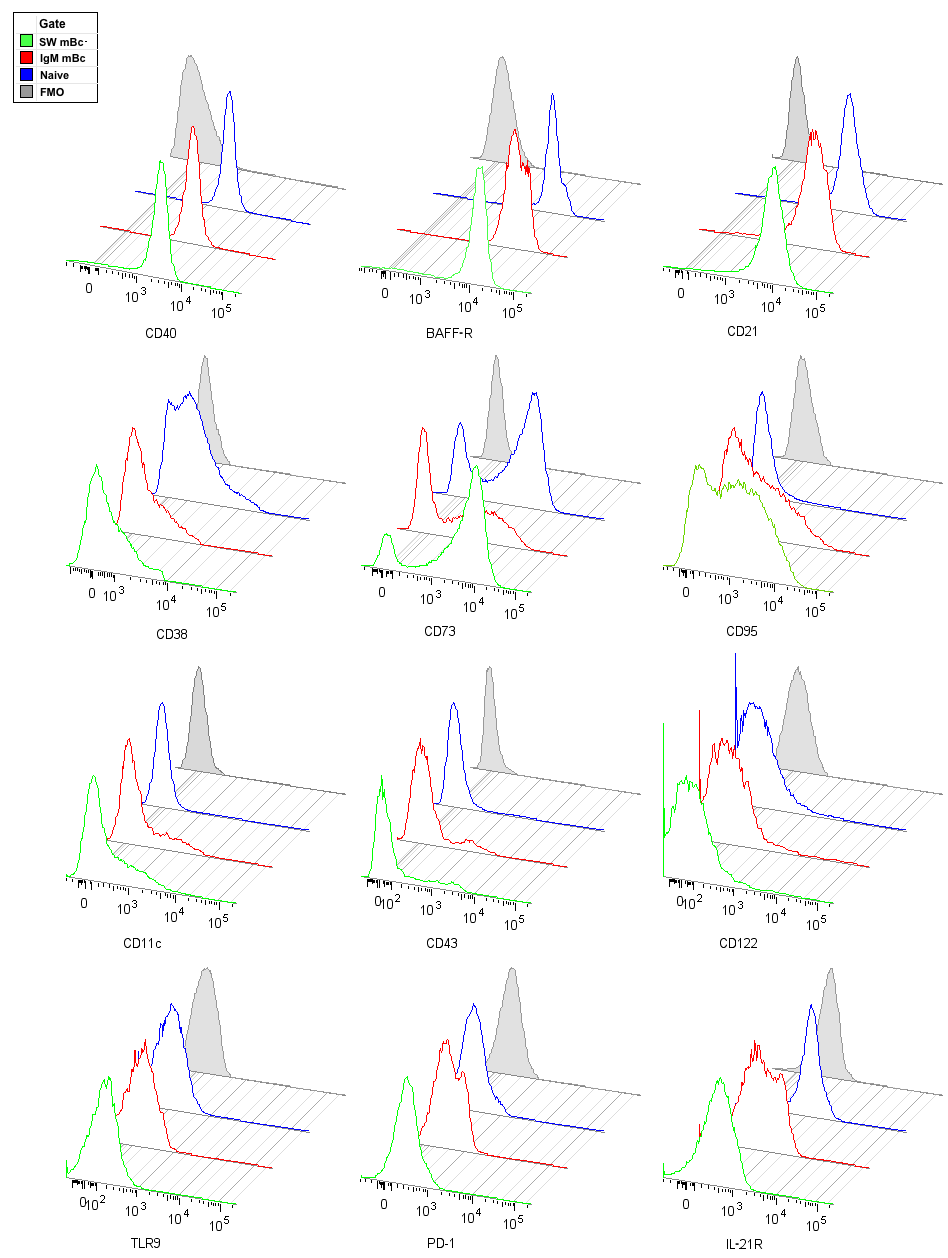

Supplement: S2 Fig — Representative histograms of the markers studied in naïve Bc (blue), IgM mBC (red) and Sw mBc (green) subsets. Solid gray histogram represents fluorescence minus one (FMO). (TIF) [file pone.0139718.s002.tif]

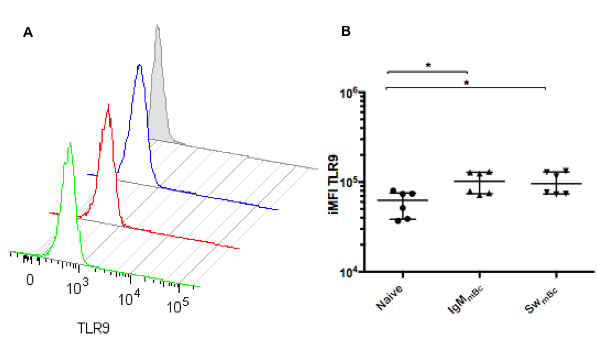

Supplement: S3 Fig — Summary of intracellular TLR9 iMFI of naïve Bc and IgM mBC and Sw mBc (n = 6). (TIF) [file pone.0139718.s003.tif]

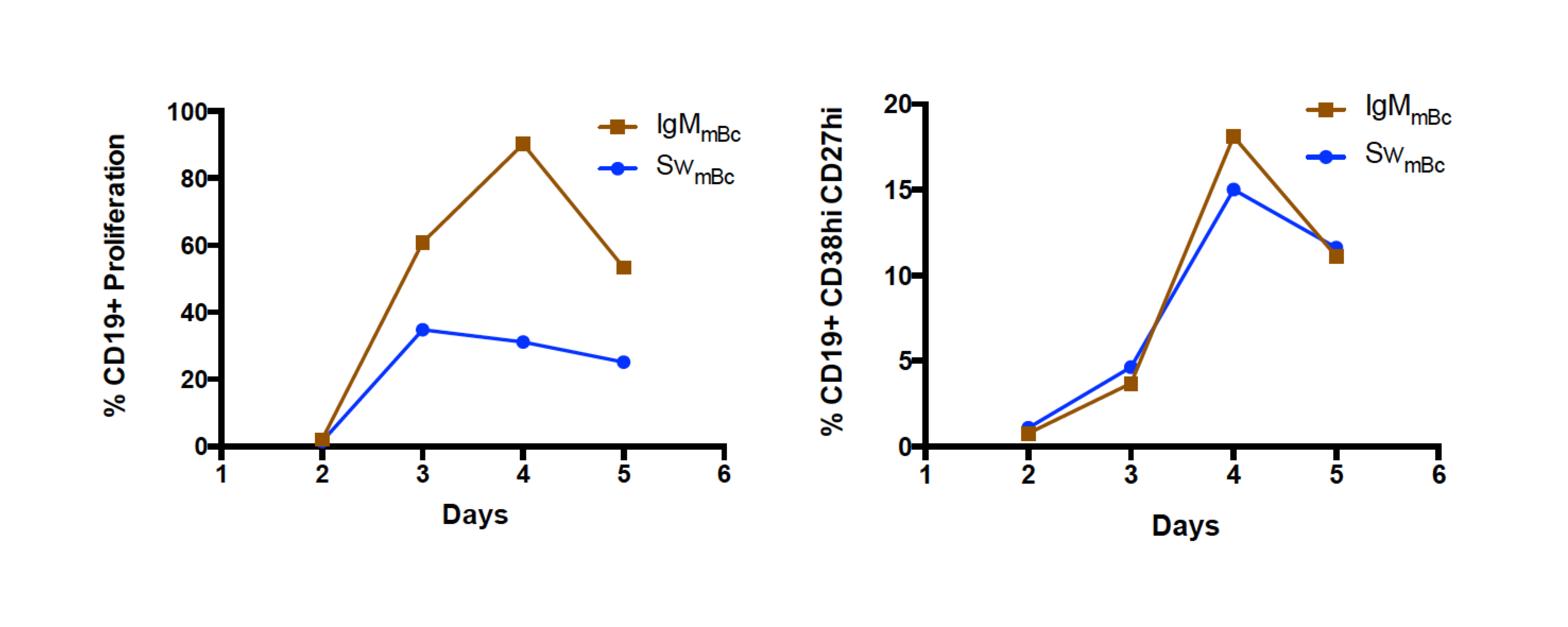

Supplement: S4 Fig — Kinetic experiment of proliferation and differentiation to ASC (CD38hi CD27hi) of IgM (brown) and switched mBc (blue) four days after stimulation with CpG, a cocktail of cytokines (IL–2, IL–6, and IL–10), and murine fibroblasts (as feeder cells) for two, three, four, and five days. Kinetic experiments for early (one, two or three days) and late responses (five, seven and ten days) were also performed using ELISPOT as readout (data not shown). Since the early time point experiments showed comparable results to the flow cytometry presented in this figure and cell mortality was above 80% in the late time point experiments (data not shown), we chose day four for the experiments reported elsewhere in this paper. (TIF) [file pone.0139718.s004.tif]

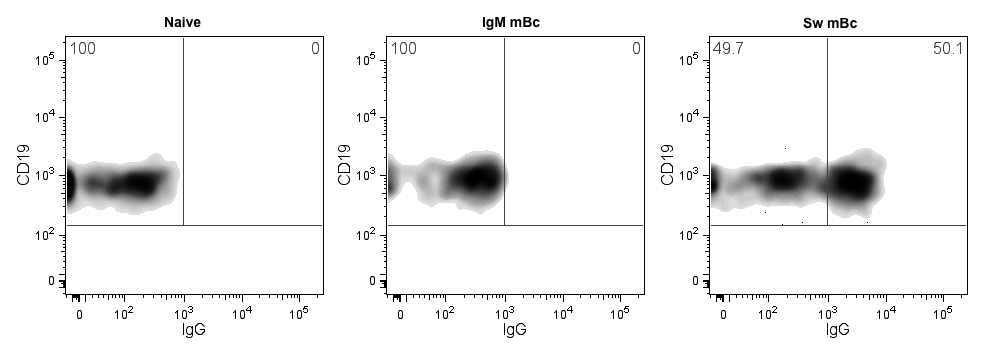

Supplement: S5 Fig — Cells from a buffy coat were sorted like for the functional experiments of Figs 3 and 4 and directly stained for intracellular IgG as described for Fig 5. (TIF) [file pone.0139718.s005.tif]
